# Inflammatory diseases of the aorta: Part 2: Infectious aortitis

**DOI:** 10.1007/s00772-016-0142-x

**Published:** 2016-06-13

**Authors:** I. Töpel, N. Zorger, M. Steinbauer

**Affiliations:** 1 Klinik für Gefäßchirurgie, KH Barmherzige Brüder Regensburg, Prüfeninger Str. 86, 93049 Regensburg, Deutschland; 2 Institut für Radiologie, Neuroradiologie und Nuklearmedizin, Krankenhaus Barmherzige Brüder, Regensburg, Deutschland

**Keywords:** Aortitis, Aortic diseases, Arteritis, Vasculitis, Infection, Aortitis, Erkrankungen der Aorta, Arteriitis, Vaskulitis, Infektion

## Abstract

Infectious aortitis is a rare but life-threatening disease. Due to impending local and systemic complications, prompt diagnosis and initiation of effective causal treatment are essential for patient survival. Differentiating infectious aortitis from other aortic diseases, in particular non-infectious aortitis, is of great importance. This article discusses the various causes, diagnostic tools, and therapeutic strategies for infectious aortitis.

## Zusammenfassung

Infektiöse Aortitiden stellen ein sehr seltenes, aber lebensbedrohliches Krankheitsbild dar. Auf Grund der drohenden lokalen und systemischen Komplikationen dieser Erkrankungen sind eine schnelle Diagnose und die Einleitung einer konsequenten kausalen Therapie essenziell für das Überleben des Patienten. Die Abgrenzung von anderen Erkrankungen der Aorta, insbesondere den nicht-infektiösen Aortitiden kommt eine große Bedeutung zu. In diesem Beitrag sollen die verschiedenen Auslöser, die diagnostischen Möglichkeiten und die Behandlungsstrategien bei infektiösen Aortitiden diskutiert werden.

## Original publication

This article is the second part of a two-part article. The first part “Inflammatory diseases of the aorta. Part 1: Non-infectious aortitis” can be found under http://dx.doi.org/10.1007/s00772-016-0143-9.

## Introduction

With the advent of antibiotics infectious aortitis became a rare occurrence. The historical term “mycotic aneurysm” was coined by Osler and describes the often mushroom-shaped presentation of aneurysms arising from infections; mycotic does not refer to a pathogenetic cause, e. g. fungal infection [35]. Left untreated, infectious inflammation of the aortic wall generally leads to aneurysm formation; however, secondary infection of a pre-existing arteriosclerotic aneurysm can also occur.

## Pathogenesis

The best known infection route is via bacteremia in the setting of pre-existing damage to the endothelium due to reduced immune barrier function. Patients with arteriosclerotic lesions of the aortic wall or arteriosclerotic aneurysms and concomitant or previous infective endocarditis, are most frequently affected [13, 40]; however, patients with a healthy aortic wall can also be affected by aortitis in the setting of endocarditis [2]. Septic embolization to the vasa vasorum and lymphogenic spread have also been described [24]. In addition to patients with arteriosclerosis, patients with congenital aortic anomalies or with generally reduced immunocompetence, such as diabetes patients, cancer patients, alcohol-dependent patients and patients receiving immunosuppressive therapy, are considered populations at risk. Alone the presence of an indwelling central venous catheter increases the risk of aortitis [24]. More rarely, pathogen invasion of the aortic wall from neighboring structures (i.e. contiguous infection) occurs (e. g. aortitis resulting from spondylodiscitis). Aortitis due to direct open contamination of the vascular wall as a result of trauma has been described in rare cases [39]. The causative focus of infection remains unclear in some patients. Thus, a number of authors have proposed classifying aortitis into primary infectious aortitis (i.e. no detectable focus) and secondary infectious aortitis (i.e. evidence of a potential focus) [10, 44].

## Microbiology

Gram-positive cocci, in particular *Staphylococcus, Enterococcus* and *Streptococcus pneumoniae*, are detected in approximately 60 % of cases of aortitis. In addition, *Salmonella* traditionally also plays an important role in the region of the abdominal aorta. Reports on numerous other bacteria acting as isolated pathogens have been published, e. g. *Listeria, Haemophilus, Bacteroides fragilis* and *Clostridium septicum* [21, 24, 38]. Although *Treponema pallidum*, the pathogen in classical syphilitic aortitis, has not played a significant role in the clinical routine since the introduction of antibiotics, the number of new cases reported in Germany has been rising since 2010 (4406 cases in 2012). Thus, the current incidence stands at 5.4 cases per 100,000 inhabitants [12].* Mycobacterium tuberculosis, Candida *and* Aspergillus* represent other rare pathogens. Spreading to contiguous areas from tuberculomas and infected lymph nodes has been described for *Mycobacterium tuberculosis* in particular [22]. An association between inflammatory vascular diseases and viral infections has also been described. The causal relationship is considered unequivocal particularly in the cases of hepatitis B and hepatitis C viral infections [43]. Vasculitis also more frequently occurs in the setting of a number of other viral infections (e.g. Epstein-Barr virus, cytomegalovirus, varicella-zoster virus and herpes simplex virus) [27], whereby it is not always clear whether processes directly caused by viruses, side effects of antiviral treatment or secondary bacterial vasculitis as a result of immune suppression play a role [15]. This applies, for example, to human immunodeficiency virus (HIV) infections, for which a wide variety of clinical manifestations of inflammatory vascular lesions have been described [37]. It was possible to detect a secondary bacterial cause in the majority of patients in whom large vessels were affected [6]. Cases of multiple mycotic aneurysms were frequently observed. Patients with no detectable bacterial infection frequently presented with multiple occlusions and aneurysms of the carotid, femoral and popliteal arteries. There was typically no evidence of arteriosclerotic lesions. Histological analysis found signs of T cell-mediated vasculitis of the vasa vasorum and periadventitial vessels, which is believed to contribute to structural instability and aneurysm formation via transient local ischemia of the vessel wall [32]. Overall, virus-related vasculitis with aortic involvement is rare [36].

## Clinical presentation

Symptoms are nonspecific and depend on the site and extent of aortitis and patients without aneurysm formation experience fewer symptoms. Clinical symptoms of the primary infection come to the fore in the case of secondary aortitis [24]. Fever combined with thoracic, abdominal or dorsal pain are the most common clinical symptoms [31]. Thoracic aortic aneurysms can additionally cause dysphagia, dyspnea, hoarseness, cough and superior vena cava compression syndrome; however, cases of entirely symptom-free patients have also been described [34].

## Diagnostic work-up

Typical laboratory findings include leukocytosis as well as elevated C‑reactive protein (CRP) levels and erythrocyte sedimentation rates (ESR). Guidelines recommend making several (≥3) sets of aerobic and anaerobic blood cultures at intervals of between 6 and 8 h prior to instigating empirical antimicrobial therapy, similar to the procedure in suspected infective endocarditis [14]. The sampling technique should fulfil established standards (Table 1) in order to prevent contamination and subsequent difficulties in the interpretation of results [16]. In cases where this approach is not possible due to the patient’s condition (e.g. septic complications, rapid deterioration of organ function and need for emergency surgery), two blood cultures taken at a 1 h interval yield a limited level of reliability [14].

**Table 1:** Blood culture sampling technique

Blood culture sets comprising 2 bottles each (1 aerobic and 1 anaerobic)
Where possible, perform sampling during fever development
Collect 2–3 sets from different puncture sites *prior* to the instigation of antibiotic therapy
Blood samples taken under ongoing antibiotic therapy should be collected immediately *prior *to administration of the next dose of antibiotics
Sampling blood from indwelling cannulas or indwelling catheters is not permitted
Disinfect bottle tops and allow to dry *fully*
Disinfect the sampling site twice *without* wiping dry
Do not palpate the puncture site again after disinfection!
Puncture the vein while wearing sterile gloves using a 20-ml syringe and a cannula (at least 20 G)
Inoculate each blood culture bottle with at least 5 ml and maximum 10 ml blood
Gently rotate blood culture bottles, do not shake (avoid foam formation)

**Fig. 1:**
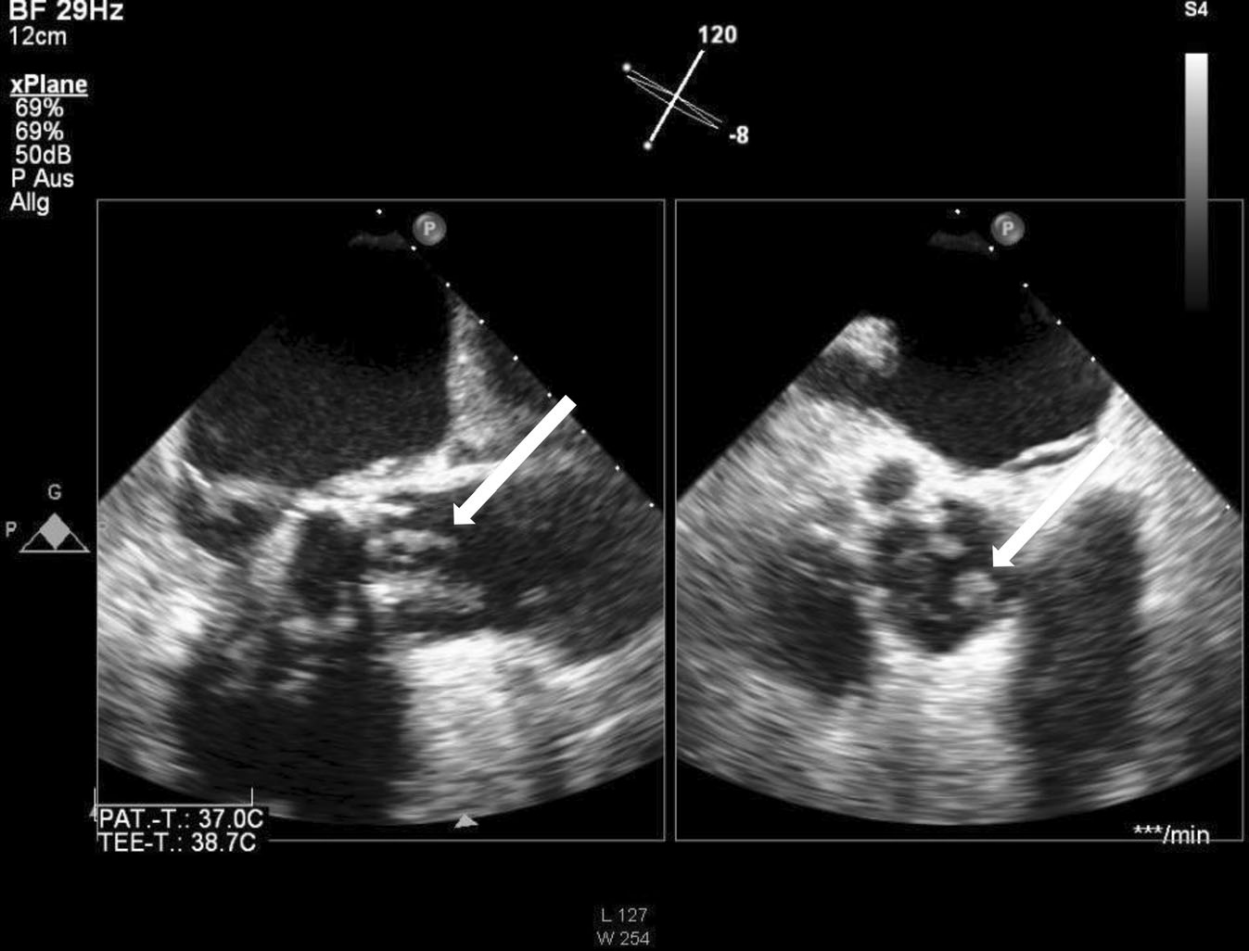
Echocardiography showing vegetations (*arrow*) in endocarditis following aortic valve repair using a bioprosthesis (courtesy of Dr. Frank Heißenhuber, Regensburg)

Blood cultures fail to detect bacteria in approximately 25 % of cases [20, 29]. In certain cases the use of a broad spectrum polymerase chain reaction test for bacteria (PCR 16 s rDNA) can reveal the identification of the pathogen [14, 25, 30]. Duplex sonography plays a key role in the region of the abdominal aorta in terms of the detection or exclusion of aortic aneurysms. Certain findings here are suggestive of aortic wall infections (e.g. the detection of gas bubbles in and around the vessel wall, vessel wall edema and perivascular fluid accumulation) [42]. Echocardiography (transthoracic transesophageal) primarily serves to exclude endocarditis. The aortic wall should be investigated for vegetation and thrombi. Aneurysms in the visible sections of the aorta are patent; however, the distal ascending aorta and the aortic arch in particular can be assessed only to a limited extent ([13, 23, 26, 40, 47]; Fig. 1).

**Fig. 2:**
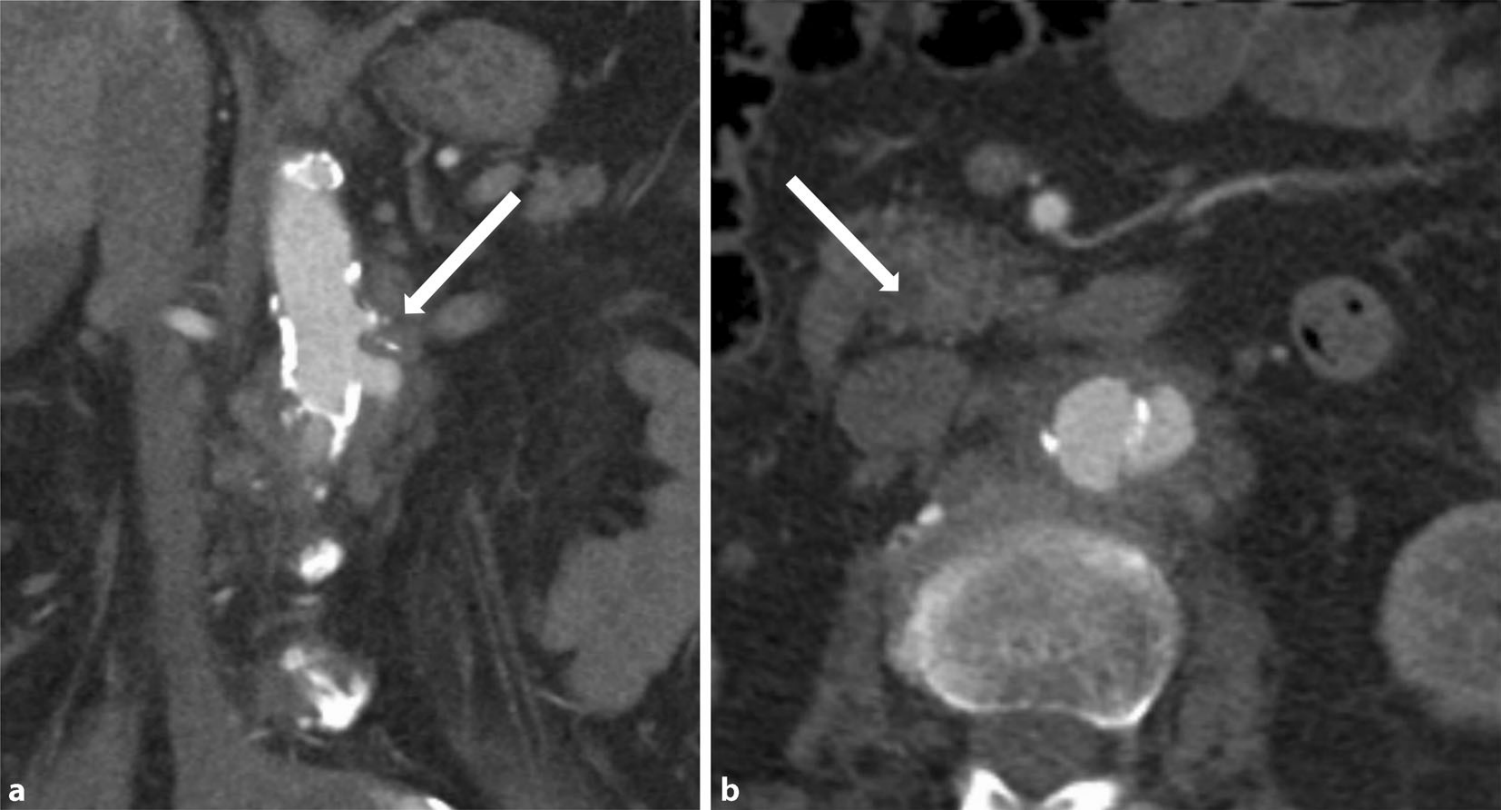
Abdominal computed tomography following intravenous contrast medium, arterial phase, coronal reconstruction. (**a**) Mycotic aneurysm of the aorta immediately below the branch of the left renal artery (*arrow*), (**b**) abdominal computed tomography following intravenous contrast medium, axial view, arterial phase. Visualization of the mycotic aneurysm with a breach in aortic wall calcification. Inflammatory wall thickening and patent inflammatory periaortic soft tissue border (*arrow*)

Computed tomography (CT) angiography with arterial and venous phase imaging enables assessment of the entire aorta. This method represents the gold standard in the diagnosis of infectious aortitis. In addition to visualization of aneurysms it also permits a precise assessment of the wall structure in a non-aneurysmal aorta. Wall thickening, contrast medium uptake in the wall (venous phase), increased perivascular streaking and fluid accumulation may be signs of vessel wall inflammation. Although gas bubbles are more rarely detected in the vascular wall, this finding has high diagnostic reliability. Rapidly progressive growth of true or false aneurysms is also suggestive of an infectious etiology ([18, 21, 24]; Fig. 2).

**Fig. 3:**
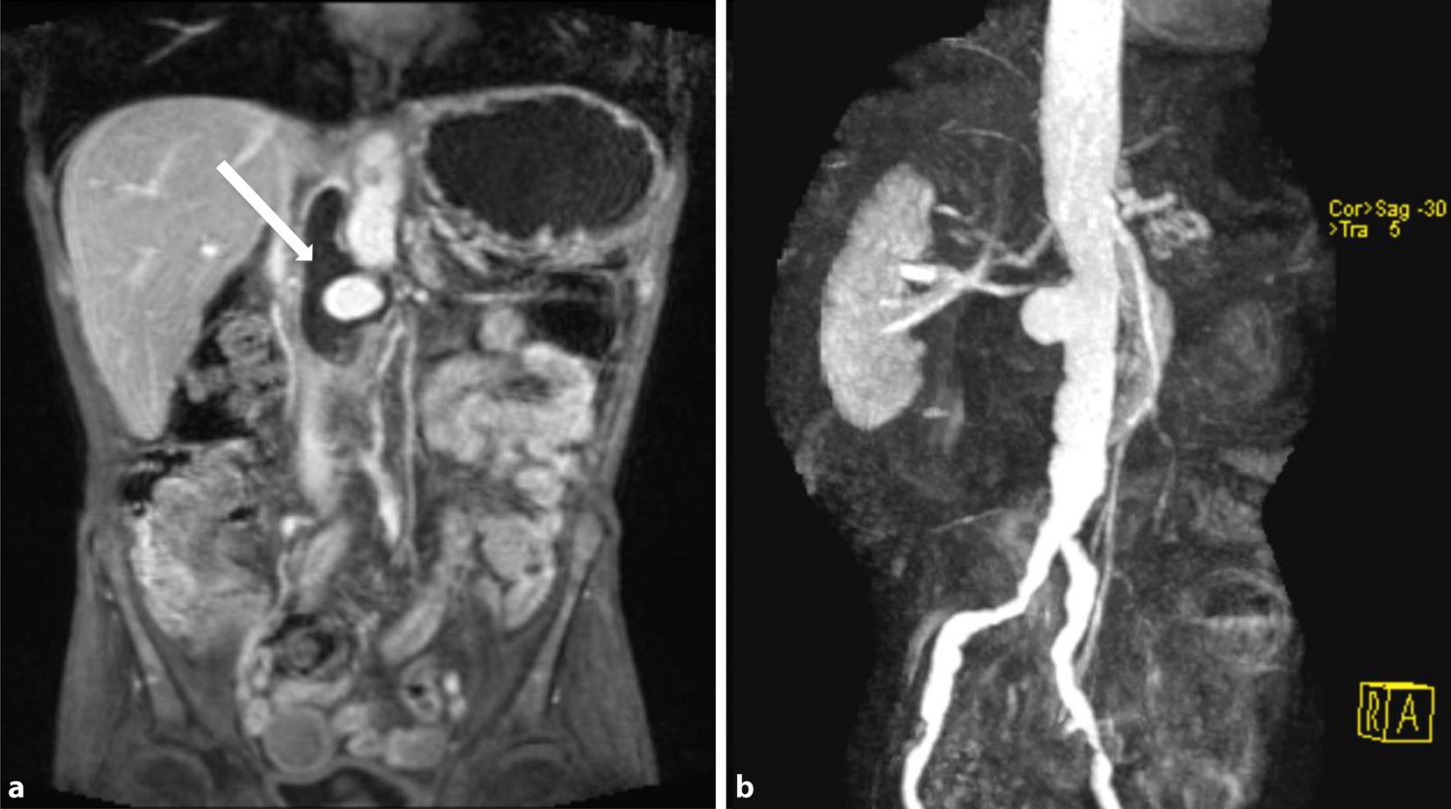
(**a**) Fast low angle shot (FLASH) magnetic resonance imaging, abdominal (T1 post-contrast medium), coronal view. Partially thrombosed, infectious false aneurysm of the abdominal aorta (*arrow*). (**b**) Magnetic resonance angiography of the abdominal aorta in the same patient. The extent of the finding as well as its inflammatory cause are distinctly underestimated in the maximum intensity projection (MIP) only reconstruction. Additional axial and coronal sequences are required in the diagnostic work-up for aortitis, in addition to magnetic resonance imaging assessment of morphology

Contrast-enhanced magnetic resonance imaging (MRI), which is technically more complex and not always available, also enables visualization of the entire aorta with good spatial resolution ([21, 39, 41]; Fig. 3); however, pulsation artefacts occur particularly in the aortic sections adjacent to the heart, thereby potentially reducing the diagnostic reliability. Fat-suppressed sequences, possibly combined with the dark-blood technique, can be used to visualize vascular wall edema more clearly [8].

**Fig. 4:**
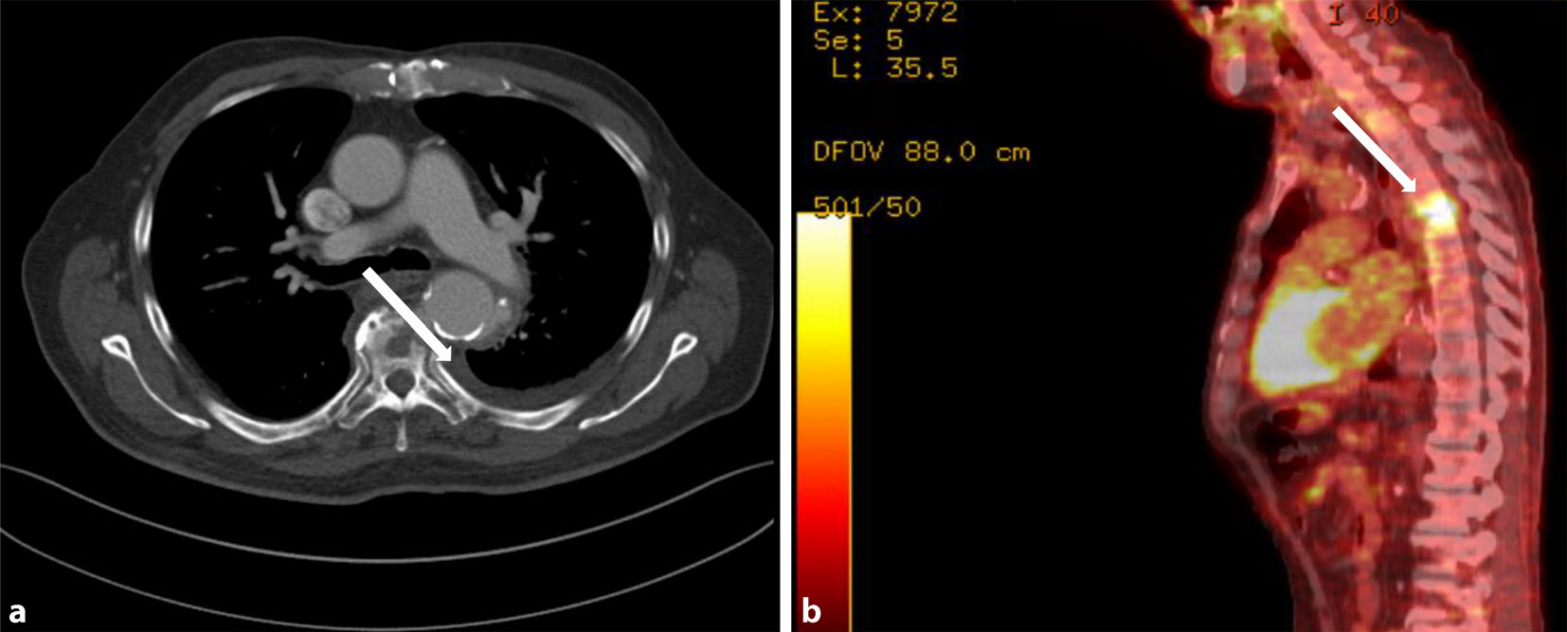
(**a**). Native thoracic computer tomography, axial view, showing mycotic aneurysm (*arrow*) of the descending aorta in direct contact to spondylodiscitis of the 4th thoracic vertebra. Additional inflammatory osteolysis of the affected vertebra. Pleural effusion left side. (**b**) Positron emission tomography fusion image, sagittal reconstruction, high activity in spondylodiscitis projecting to the 3rd and 4th thoracic vertebrae (*arrow*)

The use of ^18^F-fluorodeoxyglucose positron emission tomography-CT (FDG-PET/CT) is helpful in cases where the imaging methods previously described fail to yield unequivocal findings or where differentiation from other possible infectious foci is necessary (Treglia [4, 8, 17, 46]; Fig. 4). The FDG-PET/CT technique has now become firmly established most notably in the diagnostic assessment of patients with fever of unclear etiology [3, 9, 19].

The differential diagnostic distinction between infectious and non-infectious aortitis can be challenging in individual cases, as clinical, laboratory, radiological and nuclear medicine findings may be similar. Table 2 compares the corresponding findings in order to provide differential diagnostic guidance.

**Table 2:** Comparison of typical findings in infectious and non-infectious aortitis

	Non-infectious	Infectious
*Laboratory tests*		
C-reactive protein	↑-↑↑↑	↑-↑↑↑
Erythrocyte sedimentation rate	↑-↑↑↑	↑-↑↑↑
Leukocytes	↑-↑↑	↑-↑↑↑
Left shift		↑
Procalcitonin		↑
Blood culture	Negative	Positive in 50–75 %
*Duplex sonography*		
	Multilocular involvement of supra-aortic, mesenteric or iliac branches; homogeneous, concentric, hypoechoic wall thickening; fibrotic stenosis	Mostly one aortic section affected without inflammatory lesions in the supra-aortic, mesenteric or iliac branches; irregular, hypoechoic or hyperechoic wall thickening; evidence of false aneurysms, gas bubbles in the wall, perivascular fluid accumulation
*Computed tomography*		
	Concentric wall thickening, possibly with contrast medium uptake in the venous phase; multilocular involvement of the supra-aortic, mesenteric or iliac branches with stenosis/occlusion; ventrolaterally oriented aorta in chronic periaortitis	Concentric wall thickening, possibly with contrast medium uptake in the venous phase; mostly one aortic section affected without inflammatory lesions in the supra-aortic, mesenteric or iliac branches; irregular wall thickening, parietal thrombi, diffuse perivascular soft tissue growth and fluid accumulation, false aneurysms, gas bubbles in the vessel wall or perivascular tissue; findings of an infection focus (e. g. spondylodiscitis)
*Magnetic resonance imaging*		
	Concentric wall thickening with signal enhancement, wall edema; multilocular involvement of the supra-aortic, mesenteric or iliac branches with stenosis and occlusions; ventrolaterally oriented pannus in chronic periaortitis	Concentric wall thickening with signal enhancement, wall edema; mostly one aortic section affected without inflammatory lesions in the supra-aortic, mesenteric or iliac branches; irregular wall thickening, parietal thrombi, diffuse perivascular soft tissue growth and fluid accumulation, false aneurysms
*Positron emission tomography-computed tomography*		
	Increased activity in multiple vascular sections, no additional infection focus	Increased activity restricted to one aortic segment, additional extravasal activity focus representing an infection focus

## Treatment

The mortality rate is high among patients with infectious aortitis and if left untreated the disease is fatal. Both conservative and surgical management carry significant risks that need to be carefully weighed up on a case by case basis; however, no prospective or comparative studies are currently available [13, 24, 33]. Most authors agree that in addition to test-based systemic antimicrobial therapy, surgical excision of the (primary) infection focus and reconstruction of the affected aortic segment offer the best prospect for the complete resolution of infections [10, 24].

**Fig. 5:**
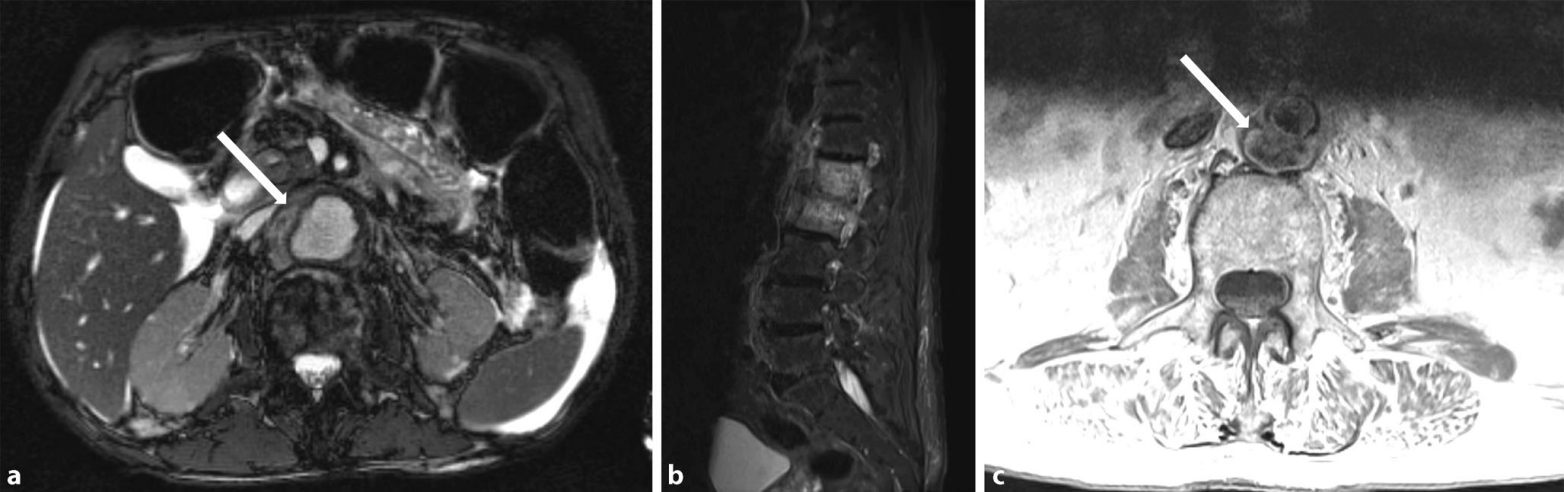
**a** Magnetic resonance imaging, abdominal (T2-weighted), axial view, marked inflammatory thickening of the abdominal aortic wall (*arrow*), perihepatic and perisplenic free fluid. **b** Significantly increased signal intensity in lumbar vertebrae 1 and 2 and intervertebral disc consistent with spondylodiscitis and prevertebral cuff of inflammatory soft tissue. **c** Inflammatory aneurysmal dilatation of the infrarenal dorsal aortic wall (*arrow*) in direct connection with the vertebral body

In the case of suspected infectious aortitis and once all diagnostic options have been exhausted, particularly detection of bacterial pathogens, intravenous antibiotic therapy should be promptly initiated, which should be adjusted and appropriately de-escalated as soon as the results of resistogram typing are available. The duration of antibiotic treatment is determined by the possibility of surgical excision of the focus, clinical and laboratory parameters, as well as by imaging findings [5, 44, 46, 48]. In primary infectious aortitis, surgical excision of the infected aortic segment also includes removal of the infection focus. In the case of secondary aortitis, excision of the infection focus is equally as important as aortic repair. It is often necessary to formulate complex interdisciplinary treatment regimens in order to ensure successful treatment for patients on a case by case basis (e. g. endocarditis and aortitis, spondylodiscitis and aortitis, Fig. 5).

Open surgical aortic repair in the presence of infection is associated with higher morbidity and mortality [10, 13]. If complications have already occurred (e.g. aneurysm rupture, false aneurysm or fistula to a hollow organ), the goal of treatment is to control hemorrhage, reconstruct the affected aortic section and remove the infection focus [10]. A one-step or multi-step approach to reconstruction can be taken depending on the individual clinical and anatomical status. Both in situ and ex situ reconstruction using autologous vein grafts, allografts and alloplastic materials have been described; however, the low case numbers in study series preclude a meaningful comparison. The choice of graft depends to a great extent on the clinical status, availability and experience of the treating team. Furthermore, intensive para-aortic debridement and biological safety measures also play an important role. Particularly in the acute hemorrhage phase, a two-step approach, comprising initial bridging by means of endovascular placement of a stent graft and secondary conversion following stabilization of the patient yields good results [28, 45]. In individual cases of primary aortitis involving less virulent bacteria that respond well to antibiotics, endovascular treatment combined with appropriate test-based antimicrobial therapy result in the resolution of infection [1, 44]. Close clinical and imaging follow-up is nevertheless necessary as persistent infection involving the stent graft is seen in approximately 20 % of patients [7, 11, 20]: therefore, when selecting a stent graft design, consideration should be given to a possible two-step graft explantation as well as the problems that might be caused by graft characteristics (e.g. hooks, barbs and clamps). Similar to the use of antibiotic-soaked Dacron stents in open aortic repair, a number of centers also use this technique for the placement of Dacron-covered endografts [44]. Scientific evidence of the efficacy of this method to reduce the rate of persistent infections involving endografts is lacking. Purely conservative treatment with antibiotics and, where necessary, surgical excision of the focus can be a promising option particularly for patients in a stable clinical condition with no acute complications and with a normal caliber aorta. Here again, close clinical and laboratory follow-up of findings as well as regular imaging are important.

## Conclusion

- Although extremely rare, infectious aortitis is associated with a high mortality rate.
- Identifying the disease and the causative infection focus form the cornerstones of successful treatment.
- With the help of modern multimodal imaging, combined with clinical presentation and laboratory and microbiological findings, it is possible to differentiate this disease entity from non-infectious inflammatory aortic disease.
- Radical surgical treatment needs to be accompanied by optimal antibiotic therapy.
- The selection of surgical approach (endovascular vs. open, one-step vs. two-step) depends on the individual case.
- A purely conservative approach is reserved for patients generally deemed inoperable or who refuse surgical treatment.
